# Analysis of Thermophysical Properties of Electro Slag Remelting and Evaluation of Metallographic Cleanliness of Steel

**DOI:** 10.3390/ma17184613

**Published:** 2024-09-20

**Authors:** Josef Walek, Adéla Odehnalová, Radim Kocich

**Affiliations:** 1 Department of Metallurgical Technologies, Faculty of Materials Science and Technology, VŠB—Technical University of Ostrava, 17. Listopadu 2172/15, 708 00 Ostrava, Czech Republic; 2 ŽĎAS, a.s., Strojírenská 675/6, 591 01 Žďár nad Sázavou, Czech Republic; adela.odehnalova@zdas.cz

**Keywords:** electro slag remelting, steel, slag, FactSage, non-metallic inclusions, metallographic cleanliness

## Abstract

Improving the competitiveness of steel companies is linked to sustainable, quality-compliant steel production. Therefore, new steel production technologies contributing to increased cleanliness of steel are continuously being developed and optimized. One way to achieve a high steel quality is to use electro slag remelting (ESR) technology. In this paper, the principle of ESR technology and the importance of fused slags for optimizing the process are outlined. The aim of this work was to analyze the main thermophysical properties of steel and fused slags used in the ESR process. Determination of the properties of steel and slags was performed using the FactSage calculation software, which involved the calculation of the liquid and solid temperature of steel and slags, the calculation and construction of quaternary diagrams, and the calculation of viscosity. The resulting quaternary diagrams revealed the substantial influence of chemical composition on melting temperatures of slags. In order to validate the acquired results, a CrNiMoV-type steel was subjected to investigation of its metallographic cleanliness and evaluation of its mechanical properties; the ESR process was shown to significantly improve the cleanliness of the steel and improve the mechanical properties of the steel compared to its cleanliness and quality when produced via vacuum degassing (VD) technology. During the ESR process, the average size of non-metallic inclusions was reduced from 20 μm to 10 μm, and the maximum size of non-metallic inclusions was reduced from 50 μm to 28 μm. The mechanical properties of the steel produced using ESR technology were impacted as follows: the ductility increased by 10%, contraction increased by 18%, notched toughness at 20 °C increased by 46%, and at −40 °C (respectively −50 °C) it increased by 30%.

## 1. Introduction

The requirements regarding the quality and durability of contemporary produced parts and components made of both steels and non-ferrous metallic materials [1,2,3,4,5] are ever increasing, which is non-negligibly related to increasing requirements regarding the cleanliness and quality of the input materials. Electro slag remelting (ESR) is a highly valuable process in steel metallurgy due to its numerous advantages over other methods [6,7,8,9,10], such as argon oxygen decarburization (AOD) [11], a ladle furnace (LF) [12], vacuum oxygen decarbonization (VOD) [13], vacuum degassing (VD) [14], and vacuum arc remelting (VAR) [15]. ESR is extensively utilized for producing high-value-added alloys, such as special steels and nickel-based superalloys. One of the key benefits of ESR is the effective elimination of non-metallic inclusions, which is crucial for enhancing the quality of the final product. This process also facilitates effective slag refining, leading to excellent solidification structures in the produced alloy. Moreover, ESR enables chemical refinement and controlled solidification, making it a preferred method for manufacturing high-performance steels [6,7,8,9,10].

The principle of ESR is in the remelting of a formed or cast metal electrode, in the herein presented case represented by a steel ingot. The electrode is gradually melted in a water-cooled crystallizer by the action of molten superheated slag. The main objective of the ESR process is to achieve higher cleanliness and the desired microstructure of the remelted metal blank. The resulting remelted ESR ingot typically features a structure with grains favorably oriented parallel to its longitudinal axis. Segregation effects are advantageously suppressed during solidification. Compared to conventionally produced ingots, an ESR ingot is homogeneous across the entire cross-section. This process enables the production of specialized products with enhanced quality, a high degree of integrity of which is required [16,17,18,19,20].

The process of steel electrode melting uses thermal energy provided by continuous resistive heating of the slag by an electric current. Due to the high electrical resistance of the slag, the passing electric current generates energy, which heats and subsequently melts the slag. The metal electrode is in contact with the liquid slag and the thermal energy of the slag gradually heats the electrode face to a temperature above the melting temperature of the metal. Melting of the metal at the electrode face is accompanied by the formation of metal droplets, which drip through the liquid slag into the metal bath. The formation of metal droplets and their passage through the molten superheated slag of a specific chemical composition is accompanied by a series of chemical reactions occurring between the metal and slag. The remelting process is performed under a protective atmosphere of an inert gas to prevent the dissolution of gases—hydrogen, nitrogen, and oxygen—into the molten slag and metal [21,22,23,24,25,26,27,28].

The slag, which is used during remelting in ESR technology, is of great importance as it is the basic compound used to melt and refine the metal. As a number of chemical and electrochemical reactions occur during the ESR process, the chemical composition and physical and physicochemical properties of the slags have to meet high requirements, including suitable specific resistance, a favorable ability to absorb non-metallic inclusions, and protection of the metal from secondary oxidation and lubrication at the interface between the copper mold and solidifying steel [29,30,31,32,33]. In order to meet the required criteria, slags for ESR must have well-defined properties. The most important parameter for ESR slags is the melting temperature, which should be lower than the melting temperature of the metal. In contrast, the working temperature must be higher than the melting point of the metal, by approximately 200–300 °C. The slags should be easier to melt because the electrode could be melted during the process when using hard-to-melt slag. In addition, relatively easy-to-melt slags facilitate degassing of the metal bath. Other important properties of slags include electrical conductivity, density, viscosity, surface tension, and emissivity [34,35].

ESR slags are mainly composed of CaF_2_, Al_2_O_3_, and MgO. In order to achieve a more homogeneous composition and compact solidification structure, the thorough removal of non-metallic inclusions is important. Inclusions readily initiate microcavities and cracks at inclusion–steel interfaces. These can subsequently act as nucleation sites for fatigue fracture or the development of other defects. The formation of non-metallic inclusions in ESR steel is influenced by many factors, including the furnace atmosphere, the content of inclusions within the melt electrode, the amount of slag and its composition, the input power, the melting rate, the filling ratio, etc. Most of the non-metallic inclusions originate from reactions between oxygen and other elements, such as manganese, silicon, and aluminum [36,37,38]. The chemical composition of the non-metallic inclusions is an important parameter influencing the mechanical properties of ESR steels. There is a marked composition difference between the oxide inclusion compositions found in ESR steels as opposed to those in conventional steels. The mechanical effects of composition differences are widely described in the literature [39,40,41].

Schneider et al. [42] discovered that the composition and type of non-metallic inclusions change significantly during remelting. Slags with a high Al_2_O_3_ content result in the presence of Al_2_O_3_-type inclusions, while slags with a low Al_2_O_3_ content result in the presence of MA spinel-type inclusions. Shi et al. [43] found that the SiO_2_ content in the CaO-Al_2_O_3_-SiO_2_-MgO inclusions was significantly reduced after the ESR process in parallel with the increase in Al_2_O_3_ content. These CaO-Al_2_O_3_-SiO_2_-MgO oxide inclusions served as precipitation sites for (Ca, Mn)S. Schnieder et al. [44] found that as a result of open remelting in the presence of air and based on changes in the activity of slag constituents, the types of non-metallic inclusions after remelting change from high-Al_2_O_3_ inclusions for slags with a high CaF_2_ content to MA spinel-type inclusions for slags with a low or no CaF_2_ content. Li et al. [45] discovered that the inclusions in the electrode were CaO-MgO-Al_2_O_3_, most of which were in the temperature range that allows the presence of non-metallic inclusions, whereas the inclusions in the ESR ingot were predominantly based on pure Al_2_O_3_. 

The removal of non-metallic inclusions in ESR occurs at the electrode tip, where non-metallic inclusions are absorbed and dissolved in the slag. As the electrode tip is heated to the melting point, the inclusions in the electrode re-dissolve before the metal melts. Any other inclusions, such as larger exogenous inclusions in the electrode, are not dissolved in the solid metal and are exposed to the slag when the electrode tip melts. If the composition of the slag is suitable, the temperature is sufficiently high, and the dwell time is sufficiently long, non-metallic inclusions will dissolve in the slag. However, further reactions may occur at this point due to differences in equilibrium constants. The flotation of large inclusions on the slag surface is also possible. At this point, the metal contains no non-metallic inclusions but can contain elements in solution that form inclusions via reactions during the solidification period. The efficiency of the removal of inclusions increases as the melting rate is reduced [46,47,48,49,50,51,52].

The aim of this study is to analyze and investigate in detail the main thermophysical properties of molten slags in the context of the ESR process. Using the FactSage computational software, the slag properties are investigated and described in detail, i.e., via a quaternary diagram, the liquid and solid temperature, the dynamic viscosity, and other important parameters. Systematic analysis revealing the effect of slag fusion on the metallurgical properties and quality of the resulting steel is performed. This comprehensive study provides a deeper understanding of the interaction between fusion slags and steel properties, and also offers valuable information and recommendations for optimizing ESR processes in industrial practice. As a result, improvements in the efficiency of production processes, as well as in the quality of the final product, can be achieved.

## 2. Materials and Methods

### 2.1. Defining the Input Data

**Figure 1:**
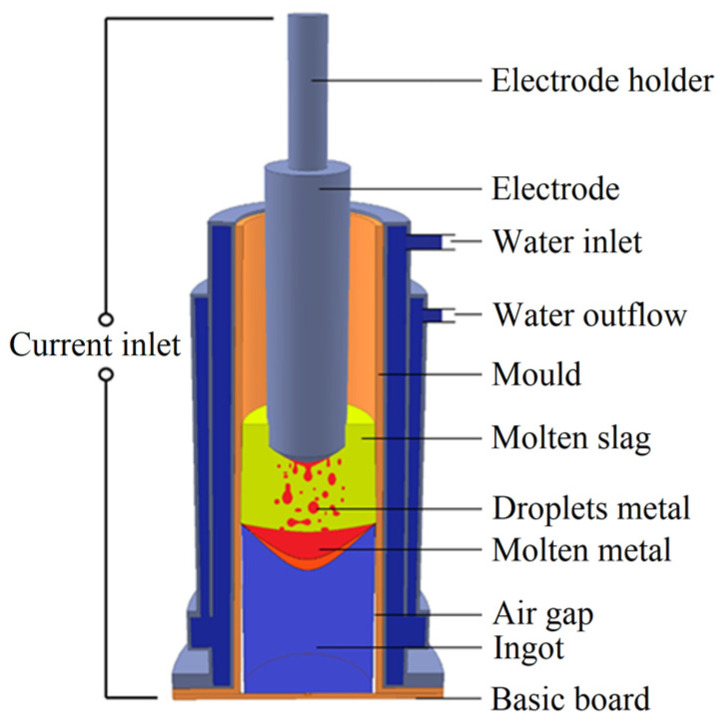
Schematic depiction of ESR device.

The experimental work was focused on determining the properties of slag and steel during the ESR process. Figure 1 shows a scheme of an ESR device. The work was performed with the FactSage software (version 8.2 by GTT-Technologies, Herzogenrath, Germany), which is one of the largest fully integrated database computer systems in chemical thermodynamics. This software is equipped with a wide range of calculation and simulation modules, and the package consists of a number of information, database, and calculation modules that access various databases of pure substances and solutions. With FactSage software, conditions for multiphase and multicomponent equilibria can be calculated with a wide variety of tabular and graphical output modes. The software is typically used, among other things, to determine the properties of steel and non-ferrous materials, such as phase transformation temperatures, austenite decomposition temperatures, liquid and solid temperatures, and others. It is also able to determine important metallurgical parameters based on chemical composition and temperature [53,54,55,56,57,58]. Since all the herein presented chemical reactions occur on the basis of thermodynamic and thermophysical events, the software is unique as it enables to study the processes of interest occurring at the slag–metal interface and to determine properties of the slag based on the acquired results.

The main components of traditional, commercially available ESR slags are usually CaF_2_, Al_2_O_3_, CaO, and MgO. Other components, added either intentionally or present as unwanted accompanying compounds and elements, were not considered in the experimental calculations. For calculations of the slag systems, slags with different ratios of the basic components within the quaternary system of CaF_2_-Al_2_O_3_-CaO-MgO were defined. In order to provide results directly applicable in the industrial practice, the exact chemical composition of the slags used as the input data for the simulations were defined based on commercially available slags by WACKER Chemistry A.G. Germany (ESR 2052, ESR 3002 ELH) and ISOMAG GmbH, Austria (AKF 235)—see Table 1 for the chemical compositions.

### 2.2. Setting the Simulation and Calculations

At first, the ESR slags’ melting intervals were determined; the chemical composition of each slag, in wt. %, from Table 1 was entered into the Equilib module. For all the slags, the compounds were selected as pure liquids and pure solids. Using the Equilib module of the software, the solid and liquid temperatures of the individual slags and mixed slags formed as mixtures of the ESR 2052 and ESR 3002 ELH slags at ratios of 2:3, 1:1, and 3:2 were calculated. The mixture of the ESR 2052 and ESR 3002 ELH slag at a ratio of 2:3 forms a complex compound consisting of 40 wt. % CaF_2_, 30 wt. % Al_2_O_3_, and 30 wt. % CaO. At a ratio of 1:1, it forms a complex compound with a content of 50 wt. % CaF_2_, 25 wt. % Al_2_O_3_, and 25 wt. % CaO. The mixture at a ratio of 3:2 then forms a complex compound containing 60 wt. % CaF_2_, 20 wt. % Al_2_O_3_, and 20 wt. % CaO. The calculations of slag melting intervals were performed in the temperature range from 1000 °C to 1450 °C, with steps of 100 °C, and the equilibrium was set as normal + transitions.

Subsequently, changes in solid and liquid temperatures of the ESR slags were determined using quaternary diagrams. Quaternary diagrams can advantageously be used to graphically express changes in the temperatures of solid and liquid of slags, depending on the chemical composition. The quaternary diagram enables the evaluation of four component systems. The studied ESR slags are three-component systems of the main compounds, namely CaF_2_, Al_2_O_3_, and CaO, with consideration of the influence of another oxide with a constant content, i.e., magnesium oxide (MgO). In the phase diagram module for plotting the quaternary diagrams, the components of interest (i.e., CaF_2_, Al_2_O_3_, CaO, and MgO) were first entered. The temperatures were selected in the range from 1200 °C to 1800 °C. The individual peaks of the diagram were determined as A—CaF_2_, B—Al_2_O_3_, and C—CaO. These conditions were set to plot all the quaternary diagrams, and the only difference was in the input contents of MgO.

Last but not least, viscosity, being an important property of ESR slags, was calculated using the viscosity module of the FactSage 8.2 software. The viscosity was calculated as temperature-dependent for all the components present in the ESR slags according to Table 1. For the ESR process, the key working temperatures of slags are in the range from 1750 °C to 1800 °C. Therefore, the viscosity calculations were performed in this specified temperature range.

Since the ESR process is very complex and its progress depends not only on the thermophysical properties of the slag but also of the remelted steels, the calculations in the FactSage software also included determination of the melting temperatures of a steel. As an example, a commercially available medium-alloyed CrNiMoV structural steel, which is typically remelted via ESR technology in real industrial conditions, was taken. The typical average chemical composition of the steel, provided by ŽĎAS, a.s. steelworks, Žďár nad Sázavou, Czech Republic, which was used as the input data for the simulations, is shown in Table 2. The individual calculation parameters were set similar to the calculation of the individual slags melting intervals. For the used structural steel, the compounds were selected as pure liquids and pure solids. Calculation of the structural steel melting interval was performed in the temperature range from 1000 °C to 1550 °C, with steps of 100 °C, and the equilibrium was set as normal + transitions. 

### 2.3. Experimental Validation and Analyses

In order to validate the calculated results, this study further involved experimental evaluation, i.e., analyses of samples from CrNiMoV structural steel forgings produced by means of VD and ESR technologies (chemical composition as stated in Table 2, provided by ŽĎAS, a.s.). For the preparation of the experimental material via ESR, the AKF 235 slag with the chemical composition as stated in Table 1 was used during remelting of the steel electrodes. Three samples of different melts from VD forgings and four samples of different melts from ESR forgings were taken for the analyses.

Firstly, the metallographic cleanliness of samples of the structural steel taken from the forgings processed using both VD and ESR technologies, according to DIN 50602 standard [59] using the K method, was evaluated. Subsequently, the samples were subjected to analysis of microstructures via scanning electron microscopy (SEM) using a JEOL device of JEOL, Ltd., Akishima, Tokyo, Japan equipped with energy-dispersive X-ray spectrometry (EDX) and electron backscatter diffraction (EBSD) detectors. Analysis of the inclusions was performed on samples taken from both the VD and ESR forgings. During the analysis, the type, fraction, and size of the present inclusions were primarily examined. Furthermore, the EDX analysis also enabled us to determine the distributions of chemical elements within the inclusions. For each sample (VD and ESR), a minimum of nine maps of distributions of chemical elements and point analyses of the chemical composition of the non-metallic inclusions, documenting their size and shape, were performed. The total of twenty-nine non-metallic inclusions from three samples from VD forgings and fifty-three non-metallic inclusions from four samples from ESR forgings were analyzed.

Last but not least, the mechanical properties of the CrNiMoV steel were evaluated to determine the influence of the production process on the quality of forgings manufactured by means of VD and ESR technologies. For the evaluation of the mechanical properties, six samples from forgings produced via VD technology and six samples from forgings produced via ESR technology were selected. The forgings from VD technology were heat-treated and quenched in oil, while forgings from ESR technology were heat-treated and quenched in water with subsequent tempering. The samples for tensile tests and notched toughness tests according to GOST V5192–78 with a U-notch were produced by means of machining.

## 3. Results

### 3.1. ESR Slags Melting Intervals

**Figure 2:**
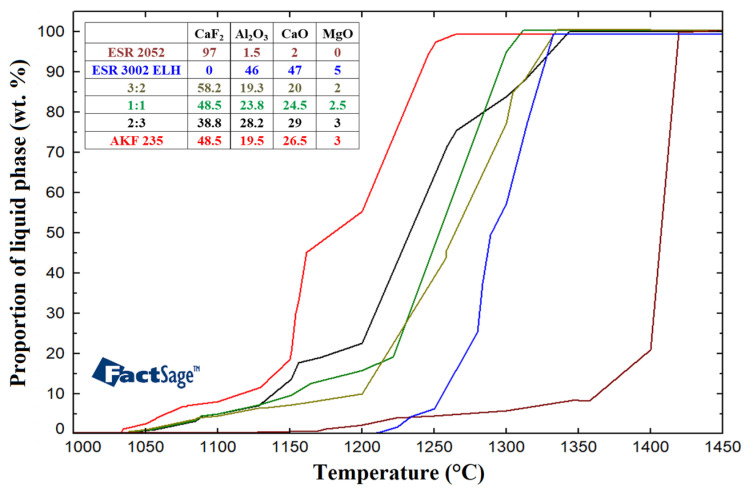
Comparison of melting temperatures for ESR slags.

The values calculated for the examined slags were subsequently compared with the values for the AKF 235 slag given in Table 1. Figure 2 shows a graphical depiction of the comparison of the melting intervals for the investigated ESR slags.

As can be seen from Figure 2, melting of the slags occurs in certain temperature intervals, and the proportions of the liquid phase increase correspondingly. From the viewpoint of the ESR process, the temperature of slag liquid and the temperature interval between the solid and liquid phases are important parameters. The melting temperature intervals are significantly influenced by the chemical compositions of the slags. The slag solid temperatures were calculated to range from 1033 °C to 1208 °C, while the liquid temperatures ranged from 1261 °C to 1419 °C. The difference between the solid and liquid temperatures for the individual slags ranged from 124 °C to 299 °C. The results of the calculations imply that the temperature intervals between the liquid and solid phases are significantly different for the monitored mixtures. This will most probably significantly affect the quality of the ingot, especially during the initial melting phase of the ESR process. The fact that a different chemical composition of a slag, e.g., with additions of TiO_2_, SiO_2_ or B_2_O_3_, can influence the liquid temperature, too, should be stated [60,61].

### 3.2. ESR Slags’ Solid and Liquid Temperatures and Quaternary Diagrams

**Figure 3:**
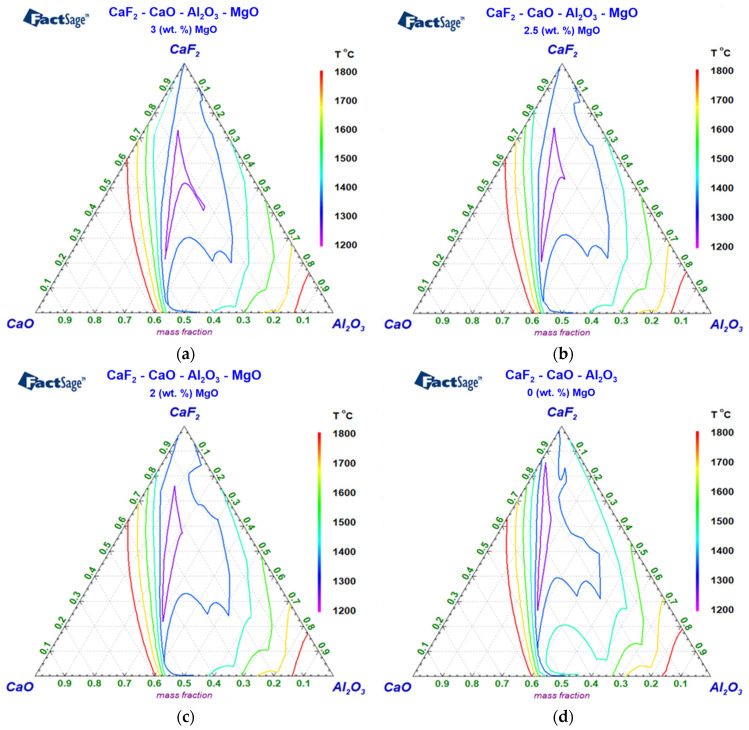
Quaternary diagrams of CaF_2_-Al_2_O_3_-CaO with (**a**) 3 wt. % MgO; (**b**) 2.5 wt. % MgO; (**c**) 2 wt. % MgO. (**d**) Ternary diagram with 0 wt. % MgO.

Quaternary diagrams of the CaF_2_-Al_2_O_3_-CaO slag system with constant MgO contents for temperatures from 1200 °C to 1800 °C are shown in Figure 3a–d, where Figure 3a shows a diagram for the CaF_2_-Al_2_O_3_-CaO slag system with 3 wt. % MgO addition, Figure 3b shows a diagram for the identical slag system with 2.5 wt. % MgO addition, Figure 3c shows a diagram for the slag system with 2 wt. % MgO addition, and Figure 3d shows a diagram for the slag without any MgO content. The colors of the curves in the diagrams depict the courses of isothermal curves for the corresponding temperature range in a selected scale. The upper temperature of the scale interval, i.e., of 1800 °C, was chosen according to the working temperatures of ESR slags in real conditions, which range from 1750 °C to 1800 °C. In technical practice, the requirements on ESR slag are mainly related to the liquid temperature of the remelted material. The ESR slag melting temperature is usually required to be about 100 °C lower than the liquid temperature of the steel. Further investigations of the slags were therefore focused on the temperature range from 1100 °C to 1450 °C.

From Figure 3a–d, we can clearly see that the concentration of MgO in a given slag system has a significant effect on both the shift and range of the melting temperature below 1400 °C. At higher melting temperatures, the effect of MgO is no longer significant. Since all the operationally used slags are fully liquid above the temperature of 1450 °C, further calculations of the quaternary diagrams and studies of the displacement of solid and liquid curves, when including the influence of MgO, were focused on the temperature range from 1100 °C to 1450 °C, with steps of 50 °C.

**Figure 4:**
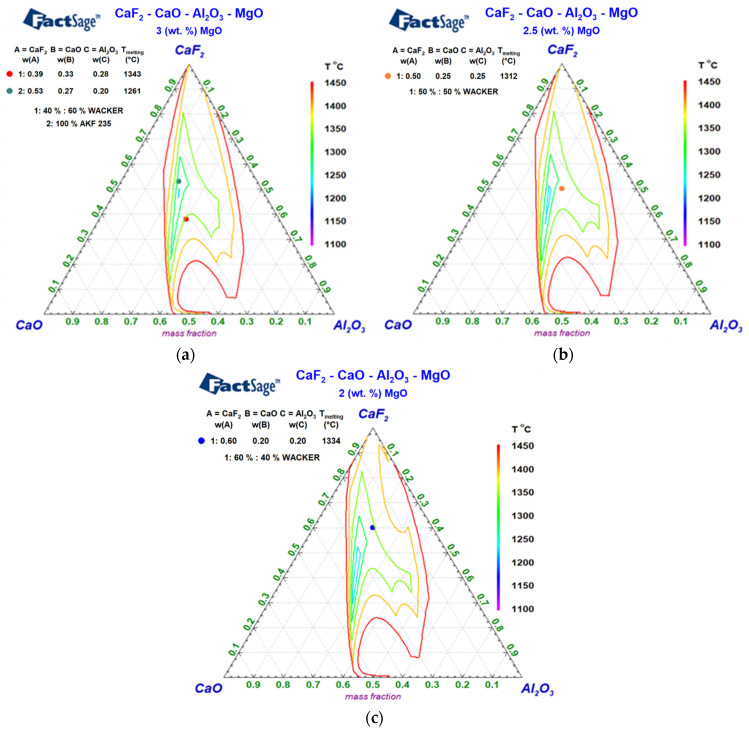
Quaternary diagram of CaF_2_-Al_2_O_3_-CaO for the slag ratios (**a**) WACKER 2:3 and AKF 235 slag; (**b**) WACKER 1:1; (**c**) WACKER 3:2.

Figure 4a–c presents the quaternary diagrams of the CaF_2_-Al_2_O_3_-CaO slag system with varying MgO contents. Figure 4a shows a CaF_2_-Al_2_O_3_-CaO diagram for the slag ratio WACKER 2:3 and AKF 235 with 3 wt. % of MgO, Figure 4b shows a diagram for the slag ratio WACKER 1:1 with 2.5 wt. % of MgO, and Figure 4c shows a diagram for the slag ratio WACKER 3:2 with 2 wt. % of MgO. The quaternary diagrams again show the melting temperature of the examined slags, the concentration of the individual slag components is mentioned in the legend.

From a detailed study of the quaternary diagrams of the CaF_2_-Al_2_O_3_-CaO slag system with varying MgO contents, we can see that the isothermal region for the temperature range from 1200 °C to 1250 °C opens and widens with decreasing MgO content. The change in MgO concentration affects the course of the isothermal boundaries of the areas, especially in the upper part of the quaternary diagram featuring higher portions of the CaF_2_ component with the lowest melting temperature of the three main components of the slag system. Above a temperature of 1400 °C, the change in MgO concentration has a negligible effect on a shift of the isothermal boundary of a melting temperature range. In Figure 4a, the points show the chemical composition of a mixture of two WACKER slags at a ratio of 2:3 and AKF 235 slag with the identical content of MgO of 3 wt. %. The point in Figure 4b shows the chemical composition of a mixture of two WACKER slags at a ratio of 1:1 with MgO content of 2.5 wt. %, and the point in Figure 4c represents the chemical composition of a mixture of two WACKER slags at a ratio of 3:2 with MgO content of 2 wt. %.

In the quaternary CaF_2_-Al_2_O_3_-CaO slag systems with varying MgO contents, all the studied slag mixtures are located in areas that are chemically suitable for usage in ESR process conditions. Significant changes in the chemical composition of the studied slag mixtures with an impact on an increase in the temperature of the liquid above 1450 °C would have a negative effect on the course of the ESR process [62].

### 3.3. Viscosity of Slags

Table 3 shows different values of dynamic viscosity of the ESR slags calculated depending on temperature. The dependence of change in the dynamic viscosity on the chemical composition of ESR slags is obvious. The given values show that the dynamic viscosity decreases as the temperature increases; a comparable effect on the dynamic viscosity change can be observed with increasing calcium fluoride content in the slag.

### 3.4. Determination of Steel Melting Temperature

**Figure 5:**
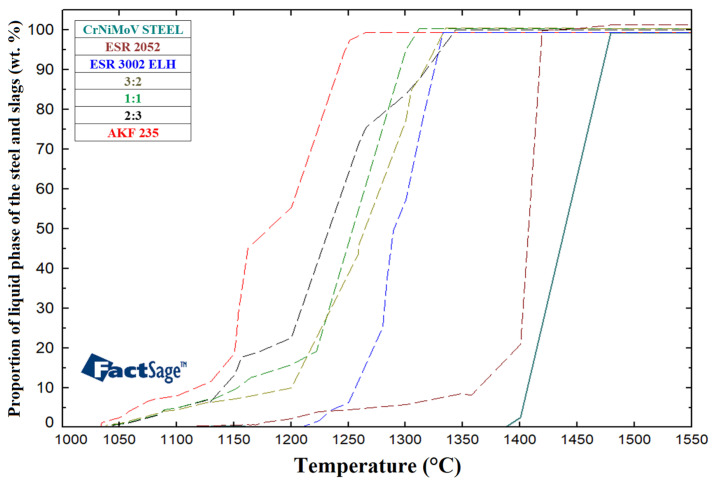
Solid and liquid temperatures for CrNiMoV structural steel.

Assessment of mutual relations between the slag and steel using the Equilib module was carried out from the point of view of solid and liquid temperatures and melting intervals of the individual components represented in the ESR process. The results are presented in Figure 5. Obviously, the steel liquid temperature of 1486 °C is significantly higher than the temperature at which the studied ESR slags consist of a 100% liquid phase. The differences between the steel liquid temperature and slag liquid temperatures are defined by the difference between the steel liquid temperature (1486 °C) and the highest slag liquid temperature (1419 °C), which is 67 °C, and the difference between the steel liquid temperature and the lowest slag liquid temperature (1261 °C), which is 225 °C.

The high melting temperatures of ESR slags (almost reaching the solid temperature of remelted steel), and therefore small differences between the steel liquid temperature and liquid temperatures of the slags, can result in deterioration of both the surface and internal quality of final ESR produced ingots. In technical practice, however, the resulting quality of an ingot is also influenced by a number of other process parameters, e.g., the rate of melting and subsequent heating of the slag in the initial phase of the ESR process.

### 3.5. Metallographic Cleanliness of Steel

Table 4 summarizes the minimum, average, and maximum values of metallographic cleanliness according to the DIN 50602 standard for forgings acquired via the VD and ESR technologies. The K method characterizes the distribution of non-metallic inclusions into a scale from K4 to K0, where K4 means the greatest contamination of steel with the largest inclusions, and K0 represents the least contamination of steel with the smallest non-metallic inclusions. After the application of ESR technology, the metallographic cleanliness of the steel is significantly improved compared to after the application of VD. Non-metallic inclusions were only classified as K2 and higher, with the maximum value of K0 = 14.1 and average value of K0 = 4.5. After the application of VD technology, non-metallic inclusions were not assigned to K3 to K0, as the steel contamination was greater and corresponded to values in the K4 scale (the highest steel contamination).

### 3.6. Evaluation of Non-Metallic Inclusions

For the examined CrNiMoV structural steel, the average size of non-metallic inclusions was calculated, and their size range was determined from all samples from forgings produced by means of VD and ESR technology. The acquired results are summarized in Table 5. The average size of the inclusions in samples from the structural steel forgings processed using VD technology was 19.8 μm, and the largest non-metallic inclusions in the respective samples from VD forgings reached a size of 50 μm. On the other hand, the average size of non-metallic inclusions for samples from the given structural steel produced by means of ESR technology was calculated to be 9.5 μm, and the maximum size of the non-metallic inclusions within the structural steel samples produced via ESR was 28 μm. In addition to the observed change in the size of the non-metallic inclusions, their morphology was also changed after the ESR technology processing. The vast majority were globular inclusions (90%), although elongated or sharp-edged inclusions were also present (10%).

**Figure 6:**
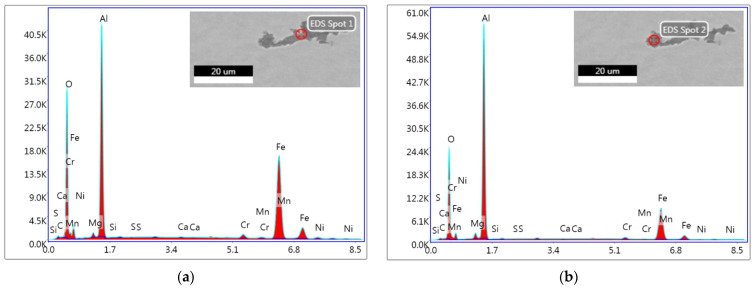
Point chemical analysis taken from structural steel produced by means of VD technology: (**a**) Al_2_O_3_ inclusion, EDS Spot 1; (**b**) Al_2_O_3_ inclusion, EDS Spot 2.

Together with electron microscopy images of the non-metallic inclusions, the presence of individual elements in the analyzed locations, marked in the images as EDS Spot 1 and EDS Spot 2, was graphically depicted. Figure 6a,b depict a large non-metallic inclusion present within a sample from the structural steel forgings produced by means of VD technology. According to the analyses performed in two individual points, as documented in the figures, this inclusion was evidently of an Al_2_O_3_ type. With its size of about 33 μm, this elongated non-metallic inclusion is among the largest found within the samples from forgings of the structural steel ingots processed by means of VD technology.

**Figure 7:**
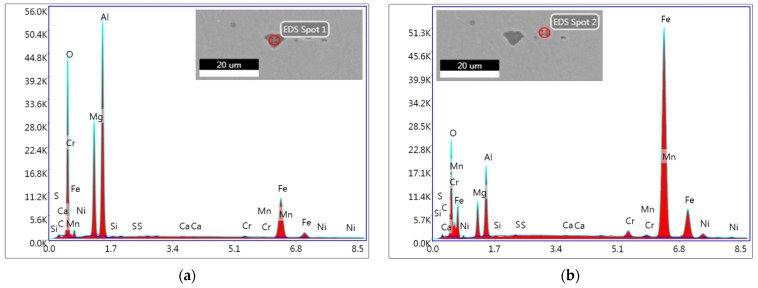
Point chemical analysis taken from structural steel produced by means of ESR technology: (**a**) Al_2_O_3_-MgO inclusion, EDS Spot 1; (**b**) Al_2_O_3_-MgO inclusion, EDS Spot 2.

Figure 7a,b show point analyses of the chemical composition of non-metallic inclusions present within the structural steel forgings produced via ESR technology. In all the samples of the structural steel (various batches), numerous non-metallic inclusions of the Al_2_O_3_-MgO type were found (see Figure 7a,b). The size, i.e., the largest dimension, of the analyzed non-metallic Al_2_O_3_-MgO inclusion shown in Figure 7a was 8 μm, and in the case of the inclusion shown in Figure 7b, its size was 5 μm. Both the non-metallic inclusions with irregular angular shapes and comparable chemical compositions were found in the same area of analysis. 

Rectangular areas comprising the examined inclusions and their surroundings were further selected for the thorough mapping of chemical composition. A total of four maps showing the distributions of the most prominent chemical elements were created for the structural steels processed by means of both the VD and ESR technologies.

**Figure 8:**
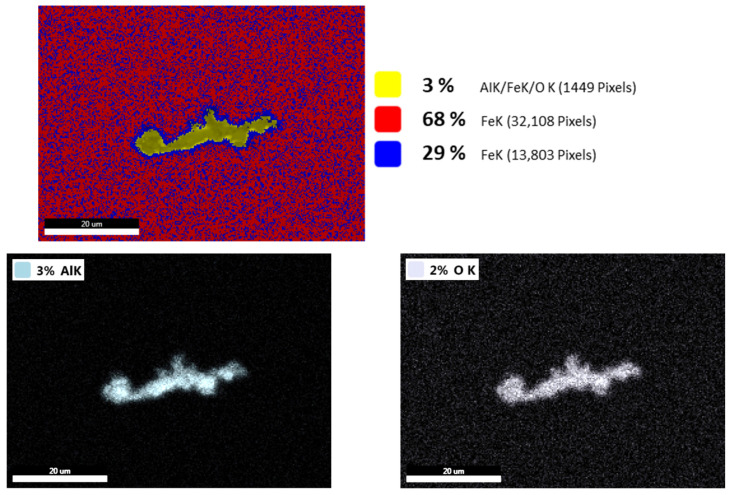
Maps of elements present in examined inclusion in structural steel produced by means of VD technology.

For the structural steel processed by means of VD technology, one of the largest non-metallic inclusions was selected for the analysis. The highest contents were detected for aluminum and oxygen, as shown in Figure 8. Based on the mutual presence of these elements, this non-metallic inclusion was most probably of an Al_2_O_3_ type. Other elements, such as calcium, manganese, chromium, or magnesium, were not detected in the inclusion.

**Figure 9:**
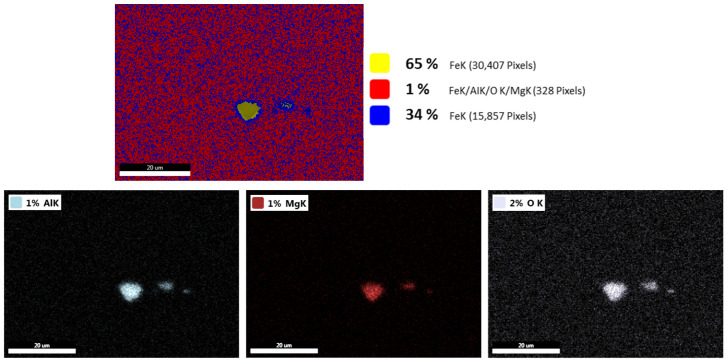
Maps of elements present in examined inclusion in structural steel produced via ESR technology.

Figure 9 shows a non-metallic inclusion that was present in the structural steel produced by means of ESR technology. As can be seen, high amounts of aluminum, magnesium, and oxygen were detected in the analyzed non-metallic inclusion, while no substantial amount of any other element was noted. According to the detected elements, it is a complex Al_2_O_3_-MgO inclusion. Non-metallic inclusions of comparable types are typically present in structural steels processed by means of ESR technology, as also confirmed by others, e.g., [63].

**Figure 10:**
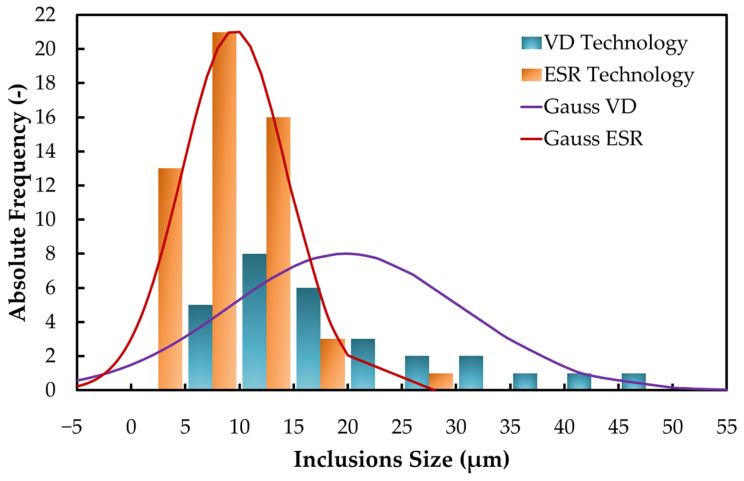
Histogram of inclusions size distribution for VD and ESR technology.

A frequency histogram was also constructed using the datasets of individual non-metallic inclusions’ size to illustrate the change in non-metallic inclusion size and the qualitative shift due to different processing technologies (VD and ESR). A histogram showing the absolute frequency per inclusions size is shown in Figure 10.

Table 6 summarizes the chemical compositions of the non-metallic inclusions detected by the analysis, and the presence of these non-metallic inclusions within the structural steel produced by means of VD and ESR technologies is compared. The occurrence of the individual non-metallic inclusions listed in this table is expressed as a representative proportion. In the structural steel processed via ESR technology, the presence of non-metallic inclusions of the Al_2_O_3_ type was not reduced when compared to the steel produced by means of VD technology. However, the occurrence of non-metallic inclusions of the SiO_2_ type was detected in the ESR steel. The occurrence of complex non-metallic inclusions of Al_2_O_3_-MgO increased significantly, but, at the same time, the fraction of complex non-metallic inclusions of Al_2_O_3_-CaO decreased. 

### 3.7. Mechanical Properties

In order to evaluate the influence of the used technology on the mechanical properties of the structural steel in depth, statistical analysis was further performed. The level of significance α was chosen as the standard 0.05, i.e., 5% unreliability of results (or 95% confidence). The statistical significance of the change in the individual mechanical properties, the *p*-value, takes on lower values in the significance level of the test than the chosen significance level α; therefore, the results are all statistically significant.

Samples for the determination of the mechanical properties of the structural steel were taken from both the head and foot of the ingots, to increase the reliability of characterization of the average mechanical properties. Six samples were taken from the head and six from the foot of the ingot, so a total of twelve tests were performed for each examined parameter. Such a high number of mechanical tests ensures the accurate determination of the average value of the mechanical properties for an ingot produced by means of VD and ESR technology.

Table 7 summarizes the results of tensile tests and notched toughness tests. Due to the higher applied rate of quenching in water, generally higher values of mechanical properties, especially strength, can be achieved (compared to conventionally produced comparable steels). Because the VD forgings exhibited larger non-metallic inclusions than the ESR forgings, the tendency of the steel produced by the VD to crack when quenched in water is relatively high. Therefore, the VD forgings were quenched in oil. The ESR forgings, on the other hand, were quenched in water as the occurring non-metallic inclusions were smaller, which finally provided the steel produced by means of ESR technology with more favorable mechanical properties.

Table 7 shows that, for the structural steel produced by means of ESR technology, the values of all the measured mechanical properties were higher than those acquired for the steel produced by means of VD technology. The tensile strength increased by up to 4.2%, ductility increased by 9.8%, contraction increased by 17.7%, notched toughness at 20 °C increased by 45.5%, and notched toughness at −40 °C (respectively −50 °C) increased by up to 29.7%. Although the strength of the ESR steel increased, most probably due to the use of a different quenching medium, no evident decrease in the plastic properties was observed. This demonstrates the positive effect of ESR technology on the mechanical and utility properties of the produced steel. In order to further enhance the performance of the produced steels, as well as to fabricate required workpieces or final products, processing via modern methods of plastic deformation can advantageously be applied [64,65,66,67,68].

### 3.8. Fractography

**Figure 11:**
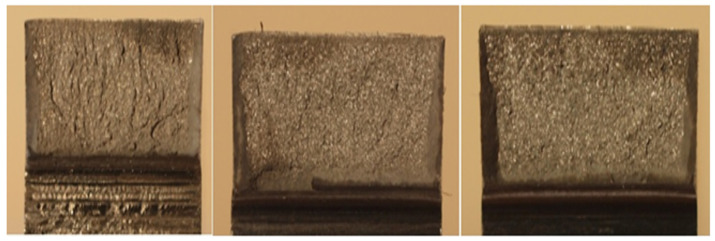
Evaluation of fractured surfaces of notched toughness samples for steel produced by means of VD technology.

**Figure 12:**
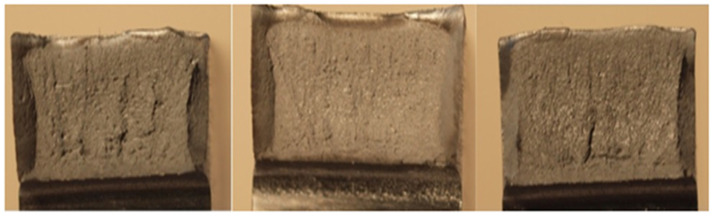
Evaluation of fractured surfaces of notched toughness samples for steel produced by means of ESR technology.

Last but not least, the fracture surfaces of the samples after the notched toughness test according to GOST V5192–78 were evaluated. Typical examples of fracture surfaces of the examined steel samples of produced by means of VD and ESR technologies are shown in Figure 11 and Figure 12, where Figure 11 shows the fractures for the VD steel and Figure 12 shows the fractures for the ESR steel. The fracture surfaces of the 10 × 10 mm samples were evaluated. The samples taken from forgings produced by means of ESR technology exhibited in 95% of cases the occurrence of fractures of the first grade, i.e., 100% ductile fracture of a gray color, with evident plastic deformation resulting in destruction of the sample. The fractured samples taken from forgings produced by means of VD technology were classified as grade four—fractures with crystalline shiny sections occupying more than 50% of the fractured surface. These results are consistent with the evaluations of strength and plastic properties of the steel.

### 3.9. Discussion of Results

In the production of structural components from high cleanliness steels, the metallographic cleanliness of the steel must match the quality of the tertiary metallurgy. The performance analyses and comparison of materials produced by secondary metallurgy (EAF-LF-VD) and materials subsequently processed by via tertiary ESR technology (EAF-LF-VD-ESR) revealed differences between the individual materials in terms of metallographic cleanliness and mechanical properties achieved. 

Basic analyses of standard slag systems have shown differences in the chemical composition of the slags. The chemical composition of the slag has a significant effect on the physical properties of the melting slag in the ESR process. In the ESR process, the slags contribute significantly to the progress of the melting process. In addition to the basic process parameters of melting rate and power consumption, slags influence the chemical composition of the remelted steel and the original non-metallic inclusions. The consequences of the interaction between the molten metal and the slag must be considered in a wider context. Based on a detailed knowledge of slag and steel properties, it is possible to study and define the causes of changes in the morphology of non-metallic inclusions with a direct continuity to the parameters of the utility properties of the steel.

The results of the metallographic cleanliness measurements of the steel confirmed the assumption of a significant improvement in the micro-cleanliness of the steel evaluated according to DIN 50602. In the steel processed via ESR technology, the value of the average inclusions size was reduced. In terms of chemical composition and the occurrence of non-metallic inclusions, the number of Al_2_O_3_-CaO based inclusions was reduced by the ESR technology remelting, and new Al_2_O_3_-based MgO enriched inclusions were now found in the steel. In most cases, non-metallic inclusions have a negative effect on the properties of the steel. The objective is to achieve a quantity, chemical composition, shape, and arrangement of inclusions in the steel that have the least impact on the desired properties. The application of ESR technology resulted in a significant change in the morphology of the inclusions, which had a positive effect on the mechanical properties achieved. In terms of mechanical properties, the steel processed via ESR technology has higher values of ultimate strength, yield strength and simultaneously higher toughness. The fracture surfaces of the notched toughness test samples for the EAF-LF-VD and EAF-LF-VD-ESR steelmaking processes compared showed fractographic differences in fracture initiation and propagation.

The presence of non-metallic inclusions of Al_2_O_3_-MgO, according to the knowledge obtained, results from the presence of MgO in commercially available slags intended for ESR processes. From the point of view of the ESR process, the variation of the MgO content in the slags are important parameters of the slag. From the results of simulations of quaternary diagrams of the CaF_2_-Al_2_O_3_-CaO-MgO slag system, it was observed that the change in MgO concentration in the slag in the range of 0 to 3 wt. % MgO leads to a change in the liquid temperature and, therefore, a change in the viscosity of the molten slag can be assumed depending on the temperature and MgO content.

## 4. Conclusions

The aim of this study was to analyze and investigate in detail the main thermophysical properties of selected molten slags used for the ESR process; the properties of a steel produced by means of ESR technology were also compared to those of a comparable steel produced by means of VD technology. The positive effect of tertiary metallurgy on the performance of the final product was clearly demonstrated. ESR technology contributes significantly to cleanliness of the steel, especially in terms of the size of the occurring non-metallic inclusions. The average size of inclusions was reduced from 20 μm to 10 μm, and the maximum size of inclusions was reduced from 50 μm to 28 μm (when compared to VD technology). In terms of the chemical composition of the inclusions, a significant reduction in the occurrence of Al_2_O_3_-CaO and Al_2_O_3_-CaS-CaO inclusions was observed. The mechanical properties of the steel produced by means of ESR technology significantly exceeded those acquired for the steel produced using VD technology alone. Even after modifying the heat treatment regime, the achievement of higher values of strength went hand in hand with a significant increase in the plastic properties of the steel produced by means of ESR technology. The ductility increased by 10%, contraction increased by 18%, notched toughness at 20 °C increased by 46%, and that at −40 °C (respectively −50 °C) increased by 30% when compared to VD technology. Although this study is performed in cooperation with the ŽĎAS, a.s., commercial company, the achieved results are versatile and can be applied directly in real industrial environments of similar commercial facilities.

## Figures and Tables

**Table 1:** Chemical compositions of commercially available ESR slags used for simulations (wt. %).

**ESR 2052**											
CaF_2_	Al_2_O_3_	CaO	MgO	SiO_2_	TiO_2_	FeO	H_2_O	C	P	S	Pb
97	1.5	2	0	0.5	0	0.2	0.005	0.03	0.005	0.03	0.0002
**ESR 3002 ELH**											
CaF_2_	Al_2_O_3_	CaO	MgO	SiO_2_	TiO_2_	FeO	H_2_O	C	P	S	Pb
0	46	47	5	0.8	0.2	0.3	0.005	0.03	0.005	0.05	0
**AKF 235**											
CaF_2_	Al_2_O_3_	CaO	MgO	SiO_2_	TiO_2_	FeO	H_2_O	C	P	S	Pb
48.5	19.5	26.5	3	1	0.3	0.5	0.05	0.1	0.05	0.05	0.005

**Table 2:** Chemical composition of the used CrNiMoV structural steel (wt. %).

C	Mn	Si	P	S	Cr	Ni	Cu	Mo	V
0.37	0.40	0.27	0.007	0.004	1.37	3.30	0.14	0.40	0.14

**Table 3:** Results of calculations of dynamic viscosity of ESR slags.

Slag	ESR 2052 and ESR 3002 ELH						AKF 235	
Ratio	2:3		1:1		3:2		1	
Temperature (°C)	1750	1800	1750	1800	1750	1800	1750	1800
Viscosity (Pa·s)	0.017	0.015	0.014	0.012	0.011	0.009	0.013	0.011

**Table 4:** Comparison of metallographic cleanliness of the structural steel according to DIN 50602, K method.

DIN 50602		K0	K1	K2	K3	K4
VD technology	minimum	-	-	-	-	0
	average	-	-	-	-	6.1
	maximum	-	-	-	-	12.5
ESR technology	minimum	0	0	0	0	0
	average	4.5	1.6	0.5	0	0
	maximum	14.1	6.8	4.9	0	0

**Table 5:** Size range and average size of non-metallic inclusions in examined structural steel.

Size of the Non-Metallic Inclusions		
(μm)	VD Technology	ESR Technology
minimum	8	2
average	19.8	9.5
maximum	50	28

**Table 6:** Representative proportion of inclusions of various types in the structural steels.

Composition of the Inclusions	VD Technology	ESR Technology
Al_2_O_3_	21	21
Al_2_O_3_-MgO	7	49
Al_2_O_3_-CaO	45	13
Al_2_O_3_-MgO-CaO	10	8
Al_2_O_3_-MgO-CaS-CaO	3	2
MgO	0	2
Al_2_O_3_-MgO-CaS	3	0
CaS	0	2
Al_2_O_3_-CaS-CaO	10	0
SiO_2_	0	2
CaS-CaO	0	2

**Table 7:** Evaluation of mechanical properties of produced structural steels.

**VD Technology**																
**Casting**	**Ru** **(MPa)**		**Rp_0.2_** **(MPa)**		**Rm** **(MPa)**		**A5** **(%)**		**Z** **(%)**		**KCU/20 °C** **(J·cm^2^)**			**KCU/−40 °C** **(J·cm^2^)**		
1	1157	1146	1309	1312	1436	1443	12.1	12.1	42.5	43.5	34	34	34	28	32	30
2	1143	1146	1330	1337	1457	1461	11.3	11.3	43.8	38.9	34	36	35	28	30	32
3	1178	1185	1348	1351	1468	1468	9.8	10.0	42.5	41.7	30	28	29	30	26	28
4	1185	1196	1348	1341	1479	1482	11.2	9.0	45.5	34.9	28	30	29	28	28	28
5	1196	1181	1362	1341	1482	1461	9.7	9.7	40.2	37.0	30	28	29	30	26	28
6	1150	1164	1326	1326	1447	1450	11.2	11.2	44.3	41.2	32	30	31	32	30	31
Ø	1169		1336		1461		10.7		41.3		31.2			29.2		
**ESR Technology**																
**Casting**	**Ru** **(MPa)**		**Rp_0.2_** **(MPa)**		**Rm** **(MPa)**		**A5** **(%)**		**Z** **(%)**		**KCU/20 °C** **(J·cm^2^)**			**KCU/−40 °C** **(J·cm^2^)**		
1	1249	1269	1359	1351	1488	1474	12.4	13.0	50.8	52.0	46	48	47	40	38	39
2	1280	1255	1416	1398	1548	1541	12.0	12.2	49.5	51.6	46	48	47	38	36	37
3	1266	1273	1406	1409	1542	1542	11.4	10.3	49.6	50.9	38	38	38	36	32	34
4	1259	1218	1404	1415	1504	1518	9.7	14.0	41.9	53.3	54	54	54	46	50	48
5	1278	1292	1360	1380	1490	1502	11.4	11.2	46.7	46.3	44	44	44	36	34	35
6	1268	1275	1446	1421	1571	1555	11.9	11.7	44.3	46.7	42	42	42	34	34	34
Ø	1265		1397		1523		11.8		48.6		45.3			37.8		
Difference (%)	+8.2		+4.6		+4.2		+9.8		+17.7		+45.5			+29.7		
*p*-value	0		3 × 10^−13^		5 × 10^−12^		5 × 10^−4^		1 × 10^−12^		0			6 × 10^−13^		

## Data Availability

The raw/processed data required to reproduce these findings cannot be shared at this time due to technical or time limitations.
